# A novel interface between the N-terminal and coiled-coil domain of STAT1 functions in an auto-inhibitory manner

**DOI:** 10.1186/s12964-023-01124-1

**Published:** 2023-07-10

**Authors:** Linus Remling, Anke Gregus, Oliver Wirths, Thomas Meyer, Julia Staab

**Affiliations:** 1 Department of Psychosomatic Medicine and Psychotherapy, University Medical Center Göttingen, Göttingen, Germany; 2 Department of Psychiatry and Psychotherapy, University Medical Center Göttingen, Göttingen, Germany; 3 German Centre for Cardiovascular Research (DZHK), Partner Site Göttingen, Göttingen, Germany; 4 Department of Psychosomatic Medicine and Psychotherapy, Laboratory of Molecular Psychocardiology, University of Göttingen, Waldweg 33, 37073 Göttingen, Germany

**Keywords:** STAT signaling, Cytokine response, Signal transduction, Interferon signaling, Gene expression

## Abstract

**Background:**

STAT1 is an intracellular signaling molecule that is crucially involved in the regulation of the innate immune system by activation of defense mechanisms against microbial pathogens. Phosphorylation-dependent activation of the STAT1 transcription factor is associated with a conversion from an antiparallel to parallel dimer configuration, which after nuclear import binds to DNA. However, not much is known about the specific intermolecular interactions that stabilize unphosphorylated, antiparallel STAT1 complexes prior to activation.

**Results:**

In this study, we identified a previously unknown interdimeric interaction site, which is involved in the termination of STAT1 signaling. Introduction of the glutamic acid-to-alanine point mutation E169A in the coiled-coil domain (CCD) by site-directed mutagenesis led to increased tyrosine phosphorylation as well as accelerated and prolonged nuclear accumulation in transiently transfected cells. In addition, DNA-binding affinity and transcriptional activity were strongly enhanced in the substitution mutant compared to the wild-type (WT) protein. Furthermore, we have demonstrated that the E169 residue in the CCD mediates the release of the dimer from the DNA in an auto-inhibitory manner.

**Conclusion:**

Based on these findings, we propose a novel mechanism for the inactivation of the STAT1 signaling pathway, assigning the interface with the glutamic acid residue 169 in the CCD a crucial role in this process.

Video Abstract

**Supplementary Information:**

The online version contains supplementary material available at 10.1186/s12964-023-01124-1.

## Background

Signal transducer and activator of transcription 1 (STAT1) plays a crucial role as a transcription factor in many cellular processes including differentiation, inflammation, and apoptosis [1–3]. Upon activation through cytokine binding at specific receptors on the cell membrane, STAT1 is phosphorylated at a single tyrosine residue by receptor-associated Janus-activated kinases (JAKs), resulting in the formation of parallel dimers through mutual phosphotyrosine:SH2 domain interactions [2]. Activation of the JAK/STAT pathway through interferon γ (IFNγ) leads to the formation of STAT1 homodimers, termed gamma-activated factors (GAF) [4], which are then transported into the nucleus via importin-α5 [5, 6]. Here, they bind to specific DNA sequences, called gamma-activated (GAS) sites, to modulate the expression of interferon-stimulated genes (ISGs). Many of the ISGs targeted by STAT1 are involved in the regulation of the innate immune response and protect the cell from invading microorganisms.

Prior to cytokine-dependent activation, unphosphorylated STAT proteins can dimerize or exist in higher-order structures within the cytoplasm [7, 8]. It was therefore predicted that a conformational change from an antiparallel to the parallel configuration due to its tyrosine phosphorylation is one of the driving factors of STAT1 binding to DNA [9–11]. To achieve full transcriptional activity, STAT1 must be additionally phosphorylated at a serine residue in position 727 [12–14]. In fact, STAT1 knock-in mice carrying a serine 727 substitution have been shown to display a much higher susceptibility to bacterial and viral infections [15, 16].

Besides containing a nuclear export signal [17], the coiled-coil domain (CCD) of STAT1 has been attributed a role in the stabilization of antiparallel dimers through interaction with the DNA-binding domain (DBD) of a partner protomer. Several gain-of-function (GOF) mutations are located within this interface, such as F172L and R274Q, which have been identified in patients with chronic mucocutaneous candidiasis [18–23]. The increased phosphorylation and prolonged nuclear accumulation of these GOF mutants lead to an increased cellular response to cytokine activation, which impairs the natural development of T cells and increases the susceptibility to fungal infections such as chronic mucocutaneous candidiasis [24]. As many of the disease-associated mutations of STAT1 are localized in the CCD, it is evident that the CCD plays a major role in the functionality of STAT1 signal transduction.

**Fig. 1:**
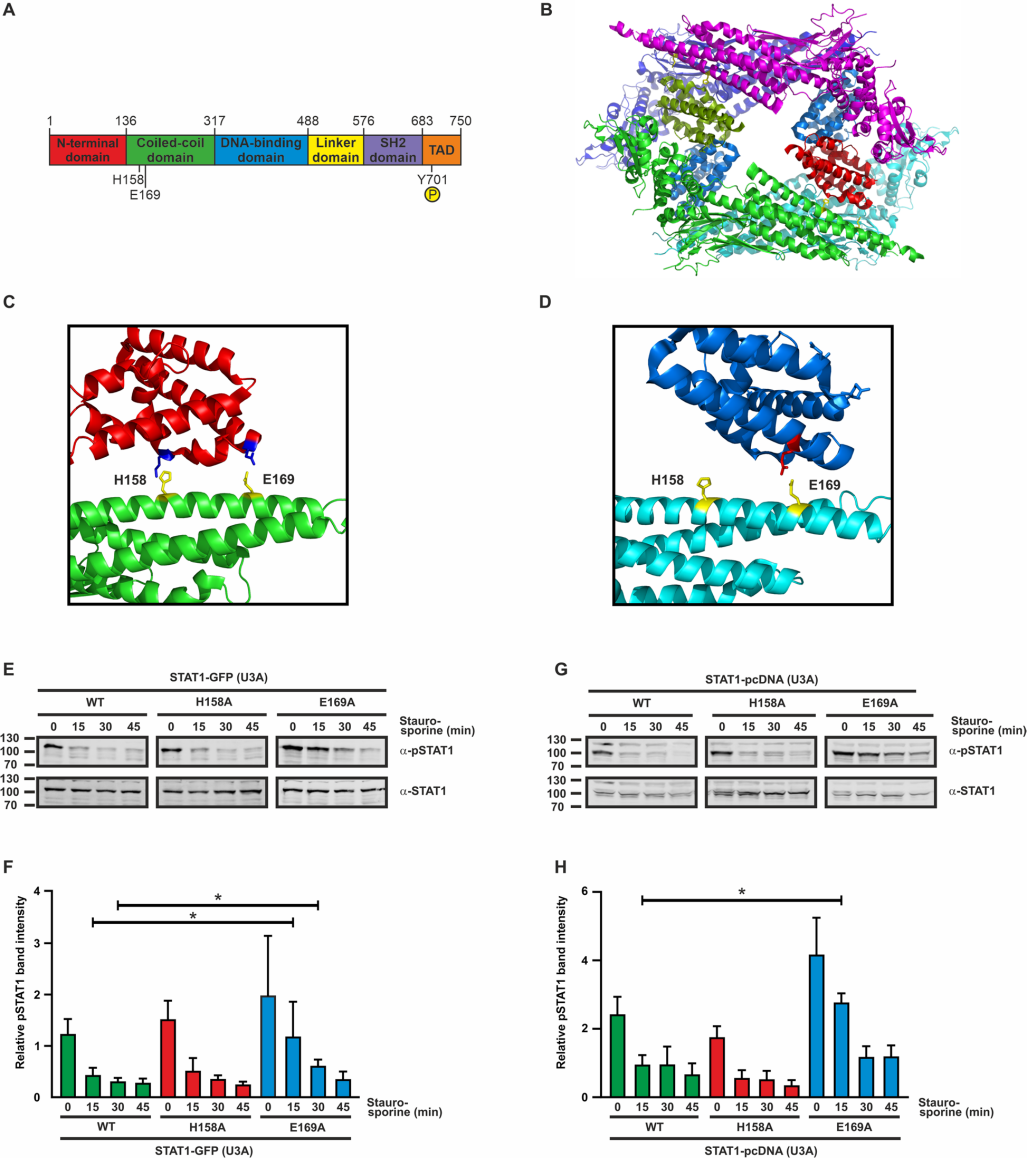
Hyper-phosphorylation of the STAT1-E169A mutant. **A** Domain structure of the STAT1 protein including the localization of residues H158 and E169 and phosphorylation site Y701. SH2 = Src-homology 2, TAD = transactivation domain.** B** Crystal structure of the STAT1 tetramer, with side chains H158 and E169 in the coiled-coil domain highlighted in yellow. Protein crystal structure renderings from the Protein Data Bank file 1YVL [25] were generated using the PyMOL software (DeLano Scientific). **C** Close-up view of the investigated interface of the front-facing protomer. Residues H158A and E169A in the coiled-coil domain (green) are marked in yellow and the putative interaction partners in the amino-terminus (red) are shown in blue. **D** The interface at the back of the tetrameric structure (as shown in **B**) between the coiled-coil domain (cyan) and the N-terminus (dark blue). **E** Representative immunoblot from whole cell extracts expressing STAT1-GFP constructs. U3A cells expressing fusions of green fluorescent protein with WT or mutant STAT1 were stimulated with 50 ng/ml IFNγ for 45 min, followed by incubation with the kinase inhibitor staurosporine (1 µM) for the indicated times. Immunoblotting was performed using phospho-tyrosine-specific (α-pSTAT1) and pan-STAT1 (α-STAT1) antibodies. **F** Quantification of immunoblotting results from three independent transfection experiments as shown in (**E**). Asterisks indicate significant difference between the WT protein and the respective mutant. **G**, **H** Representative Western blot from whole cell extracts expressing the indicated untagged STAT1 proteins (**G**), including quantification of results thereof from three independent transfection experiments (**H**)

The amino acid residues histidine 158 and glutamic acid 169 were identified as possible targets of interest to further investigate whether, under physiological conditions, they contribute to the formation of a tetrameric STAT1 complex, which was described from crystallographic data by Mao et al. [25]. These substitution sites were chosen because they are located in the CCD outside of the known interface, which involves the critical F172 residue required for the formation of an antiparallel dimer. Due to the helical arrangement of the CCD, the side chains at positions H158 and E169 point in the opposite direction as F172. Thereby, they are excluded from participation in the above-mentioned dimer interface which when disrupted is associated with the development of chronic mucocutaneous candidiasis (Fig. 1).

Interestingly, the mutation of E169 to alanine has been described in two related patients, a 53-year-old man and his 20-year-old daughter, both of whom suffered from oral and esophageal candidiasis as well as hypothyroidism, underlining the clinical relevance of this amino acid exchange [26]. Based on this observation, we hypothesized that residues H158 and E169, rather than stabilizing the dimer like other CCD amino acids, may contribute to the stability of the STAT1 tetramer and be required for fully fledged physiological functions.

## Materials and methods

### Plasmids, mutagenesis and cell culture

All experiments were conducted with HeLa cells or STAT1-negative U3A cells [27, 28]. The cells were cultured at 37 °C in a humidified 5% CO_2_ atmosphere. HeLa cells were grown in Roswell Park Memorial Institute (RPMI) 1640 medium supplemented with 10% fetal bovine serum (FBS) (Biochrom) and 100 IU/ml penicillin/streptomycin. U3A cells were cultured in Dulbecco’s modified Eagle’s medium (DMEM) (PAA Laboratories) supplemented with 10% FBS, 100 IU/ml penicillin/streptomycin and 0.04 μg/ml puromycin (Sigma-Aldrich). MegaTran2.0 (Origene) was used for transfection and, on the next day, the cells were either left untreated or stimulated with 50 ng/ml of recombinant human interferon γ (IFNγ) (Biomol) for the indicated times.

The following expression vectors were used for the transfection of HeLa and U3A cells: pEGFPN1-STAT1α (WT-GFP) coding for a fusion protein consisting of full-length human STAT1 (amino acids 1–746) and green fluorescent protein (GFP), as well as the pcDNA3.1-STAT1α vector encoding an untagged protein thereof [6]. The two vectors were also used as templates to introduce point mutations by site-directed mutagenesis using the QuikChange II kit (Stratagene), according to the manufacturer’s instructions. The following primers were used for mutagenesis at a concentration of 125 ng/µl (only forward strands are shown, mutated codons are underlined):

- H158Af; 5’–GACAAGGTTATGTGTATAGAGGCTGAAATCAAGAGCCTGG–3’, and
- E169Af; 5’–CTGGAAGATTTACAAGATGCATATGACTTCAAATGCAAAAC–3’.

All point mutations were verified by standard Sanger DNA sequencing (Seqlab).

### Protein extraction and Western blotting

Cells expressing GFP-tagged or untagged recombinant STAT1 grown on 6-well plates were lysed in 60 µl of cytoplasmic extraction buffer (20 mM HEPES, pH 7.4, 10 mM KCl, 10% (v/v) glycerol, 1 mM EDTA, 0.1 mM Na_3_VO_4_, 0.1% IGEPAL-CA-360, 3 mM 1,4-dithiothreitol (DTT), 0.4 mM Pefabloc (Sigma-Aldrich), and Complete Mini protease inhibitors (Roche)) on ice for 5 min. The cytoplasmic extracts were centrifuged at 4 °C and 16,000 g for 30 s. The supernatants were centrifuged again at 16,000 g for 5 min and collected as cytoplasmic extracts. The pellets from the first centrifugation step were resuspended in 60 µl of nuclear extraction buffer (20 mM HEPES, pH 7.4, 420 mM KCl, 20% (v/v) glycerol, 1 mM EDTA, 0.1 mM Na_3_VO_4_, 3 mM DTT, 0.4 mM Pefabloc, and Complete Mini protease inhibitors) and left on ice for 30 min. After centrifugation at 4 °C and 16,000 g for 15 min, 40 µl of the nuclear extracts were mixed with the same volume of cytoplasmic extracts from the corresponding sample to generate whole cell lysates. The cellular extracts were denatured in sodium dodecyl sulphate (SDS) sample buffer at 95 °C for 3 min and separated by 10% SDS–polyacrylamide gel electrophoresis (SDS-PAGE) with subsequent transfer onto polyvinylidene difluoride (PVDF) membranes. The membranes were blocked with 4% bovine serum albumin (BSA) in phosphate-buffered saline (PBS) for 1 h and then incubated overnight at 4 °C with rabbit monoclonal antibodies directed against either phospho-Tyr701-STAT1 (Cell Signaling Technology, 58D6) or pan-STAT1 (Cell Signaling Technology, D1K9Y), both diluted 1:1000 in blocking solution. After extensive washing, the blots were exposed to a secondary anti-rabbit antibody conjugated to IRDye 800CW (LI-COR). Bound immunoreactivity was detected using the LI-COR Odyssey imaging system.

### Fluorescence microscopy

To visualize the nuclear import of GFP-tagged STAT1, U3A and HeLa cells were grown on 8-well Lab-Tek chamber slides (Nunc). The cells were transfected and stimulated with IFNγ (50 ng/ml) for 45 min and then treated with the kinase inhibitor staurosporine (1 µM) for the indicated times. After cytokine stimulation, the cells were fixed with 4% formalin in PBS for 15 min and washed with PBS and H_2_O. The cells were then stained for 10 min with 5 µg/ml of Hoechst 33,258 (Sigma-Aldrich) and washed again.

For immunofluorescence microscopy, cells grown and stimulated in chamber slides were fixed with cold methanol at -20 °C for 15 min and subsequently washed with PBS. To avoid unspecific antibody binding, the slides were blocked with 25% FBS in PBS for 45 min at room temperature (RT), before being incubated with the α-phospho-Tyr701-specific antibody 58D6 (1:1000 in 25% FBS-PBS) for another 45 min at RT. After washing again with PBS three times, Cy3-conjugated AffiniPure anti-rabbit goat IgG (H + L) (Jackson ImmunoResearch) diluted 1:1000 in 25% FBS-PBS was used as secondary antibody for 45 min incubation at RT. After this, the cells were again washed twice with PBS and counterstained with Hoechst reagent, as described above. All samples were mounted in fluorescence mounting medium (Southern Biotech), and the intracellular fluorescence localization was visualized using a Nikon Eclipse Ti fluorescence microscope equipped with appropriate filters. Images obtained from a Nikon DS-Qi2 camera were further processed with the NIS elements (Nikon) software. Fluorescence intensities were determined both in the nucleus and cytoplasm using ImageJ (NIH), and the mean nuclear-to-total-cellular fluorescence intensities including standard deviations were calculated from 20 randomly selected transfected cells.

### Electrophoretic mobility shift assay

The DNA-binding properties of IFNγ-induced, tyrosine-phosphorylated STAT1 were assessed by electrophoretic mobility shift assay (EMSA) using several radioactively labeled DNA probes containing specific GAS elements. The following [^33^P]-labeled duplex oligonucleotide probes were created by an end-filling reaction using Klenow fragment polymerase purchased from New England Biolabs (GAS motifs are underlined):

**Table:** 

M67;	5’–CGACATTTCCCGTAAATCTG–3′,
2x GAS;	5’–CGTTTCCCCGAAATTGACGGATTTCCCCGAAAC–3′,
GAS-non-GAS;	5‘–CGTTTCCCCGAAATTGACGGATTTACCCCAAC–3‘, and
2x non-GAS;	5’–CGTTTACCCCAAATTGACGGATTTACCCCAAC–3’.

For each experiment, 4.5 µl of whole cell extracts were incubated with 8.5 µl of reaction buffer containing 1 ng of the duplex oligonucleotide probe. For competition reactions, cell lysates were incubated with [^33^P]-labeled duplex oligonucleotides in EMSA reaction buffer for 30 min on ice, and subsequently challenged by a 750-fold molar excess of unlabeled M67 DNA, for the indicated times. The samples were loaded on a 4.8% 29:1 acrylamide:bisacrylamide gel at 4 °C and separated at 400 V. DNA binding was visualized on vacuum-dried gels using the laser phosphor-imaging system Typhoon FLA 9500 (GE Healthcare Life Sciences).

### Reporter gene assay

Differential gene expression by the STAT1 mutants was examined in transfected U3A cells using reporter gene constructs. Cells were grown in 48-well plates and co-transfected with the respective STAT1 expression plasmid (250 ng), a luciferase-encoding plasmid (70 ng), and a constitutively expressed β-galactosidase plasmid (200 ng). Three different luciferase plasmids were used as reporters: 3xLy6E, pIC-339 or pIC-1352. The reporter construct 3xLy6E contains three copies of an IFNγ-inducible *Ly6E* GAS element from the promoter region of the lymphocyte antigen 6 complex locus E upstream of the transcription start site [29]. pIC-1352 is a native reporter construct containing the 1352 bp-long promoter region of the human intercellular adhesion molecule 1 (*ICAM-1*) gene or a truncated version thereof with a length of 339 bp relative to the transcription start site [30]. One day after transfection, cells were either left untreated or treated for 6 h with IFNγ, before cellular proteins were extracted with lysis buffer containing 25 mM glycylglycine, 1% Triton X-100, 15 mM MgSO_4_, 4 mM EGTA, 0.4 mM Pefabloc, 3 mM DTT, pH 7.8, and Complete protease inhibitors. Luciferase expression of the cells was assessed using the luciferase assay substrate solution from Promega in the Tecan Spark multimode microplate reader (Tecan Group) and normalized to the corresponding β-galactosidase activity, which was measured spectroscopically at 420 nm. The experiment was repeated in triplicates, and six independent transfections were tested for every combination of STAT1 variants and stimulation mode.

### Quantitative PCR

To examine the expression of endogenous STAT1 target genes, U3A cells expressing GFP-tagged STAT1 variants were cultured for 15 h in DMEM supplemented with 1% FBS, before they were either left untreated or stimulated for 6 h with 50 ng/ml IFNγ. The cellular RNA was isolated using the peqGold Total RNA kit (VWR Lifesciences), and subsequent cDNA synthesis was performed using the Verso cDNA Synthesis kit (Thermo Fisher Scientific). PCR reactions were carried out in a total volume of 20 µl, containing 25 ng of cDNA, 70 nM of each specific primer, and 10 µl of Absolute Blue qPCR SYBR Green Mix (Thermo Fisher Scientific).

The following primer pairs were used:

**Table:** 

GAPDHf	5′–GAAGGTGAAGGTCGGAGTC–′3,
GAPDHr	5′–GAAGATGGTGATGGGATTTC–′3,
STAT1f	5′–CCGTTTTCATGACCTCCTGT–′3,
STAT1r	5′–TGAATATTCCCCGACTGAGC–′3,
IRF1f	5′–AGCTCAGCTGTGCGAGTGTA–′3,
IRF1r	5′–TAGCTGCTGTGGTCATCAGG–′3,
MCP1f	5′–CCAGTCACCTGCTGTTATAAC–’3,
MCP1r	5′–TGGAATCCTGAACCCACTTCT–′3,
GBP1f	5′–GGTCCAGTTGCTGAAAGAGC–′3,
GBP1r	5′–TGACAGGAAGGCTCTGGTCT–′3,
CXCL9f	5′–CCACCGAGATCCTTATCGAA–′3,
CXCL9r	5′–CTAACCGACTTGGCTGCTTC–′3,
CXCL10f	5′–ATTCTGAGCCTACAGCAGAG–′3, and
CXCL10r	5′–GCTTGCAGGAATAATTTCAA–′3.

The PCR protocol run on an Eppendorf cycler included a denaturation step at 95 °C for 15 min, which was followed by 40 cycles of denaturation at 95 °C for 15 s, annealing at 55 °C for 30 s, and extension at 72 °C for 30 s. A melting curve analysis was performed after the final amplification step using a temperature gradient from 60 °C to 95 °C in 0.5 °C increment steps, fluorescence being measured at each temperature for a period of 10 s. All reactions were carried out in at least triplicate independent experiments. The relative expression of a transcript was normalized to the expression of *GAPDH* as determined for each sample. The ΔΔCt-method based on the formula 2^−(ΔCt target − ΔCt reference sample)^ was used to determine relative expression levels.

### Statistical analysis

Statistical analysis was performed using GraphPad Prism 9 for Windows. To determine whether differences between the groups are significant, the data were compared using Student´s *t*-test. The data are presented as mean ± standard error of the mean, with the significance threshold stated as the p-value ≤ 0.05 and marked with asterisks in the corresponding graphs. Digital images were processed by ImageJ (NIH) software and data figures were created using CorelDRAW Graphics Suite 2019.

## Results

### STAT1-E169A displays elevated tyrosine phosphorylation

In the present study, the residues E169 and the non-homologous H158 in STAT1 were mutated to alanine, and the two plasmids encoding the respective missense mutants were used in transfection experiments (Fig. 1A). In the crystal structure of the tetramer, these residues are located near the N-terminus of the same or a different protomer [25] (Fig. 1B–D). Two other members of the protein family, namely STAT3 and STAT4, similarly contain a negatively charged amino acid residue in their coiled-coil domains (D171 and E170, respectively) at the homologous position.

In transfected STAT1-negative U3A cells reconstituted with the mutants, exposure to interferon resulted in inducible tyrosine phosphorylation of the recombinant protein. When the kinase inhibitor staurosporine was used as a potent blocker of kinase activity in cells pretreated with interferon, the E169A mutant, but not H158A, showed a prolonged phase of tyrosine phosphorylation (Fig. 1E, F, Supplemental Fig. 1). Similar results were obtained when U3A cells were transfected with plasmids coding for the untagged constructs instead of the GFP-tagged variants (Fig. 1G, H). Increased tyrosine phosphorylation was also observed when the experiment was repeated in transfected HeLa cells (data not shown).

### Tyrosine-phosphorylated STAT1-E169A shows prolonged nuclear accumulation

**Fig. 2:**
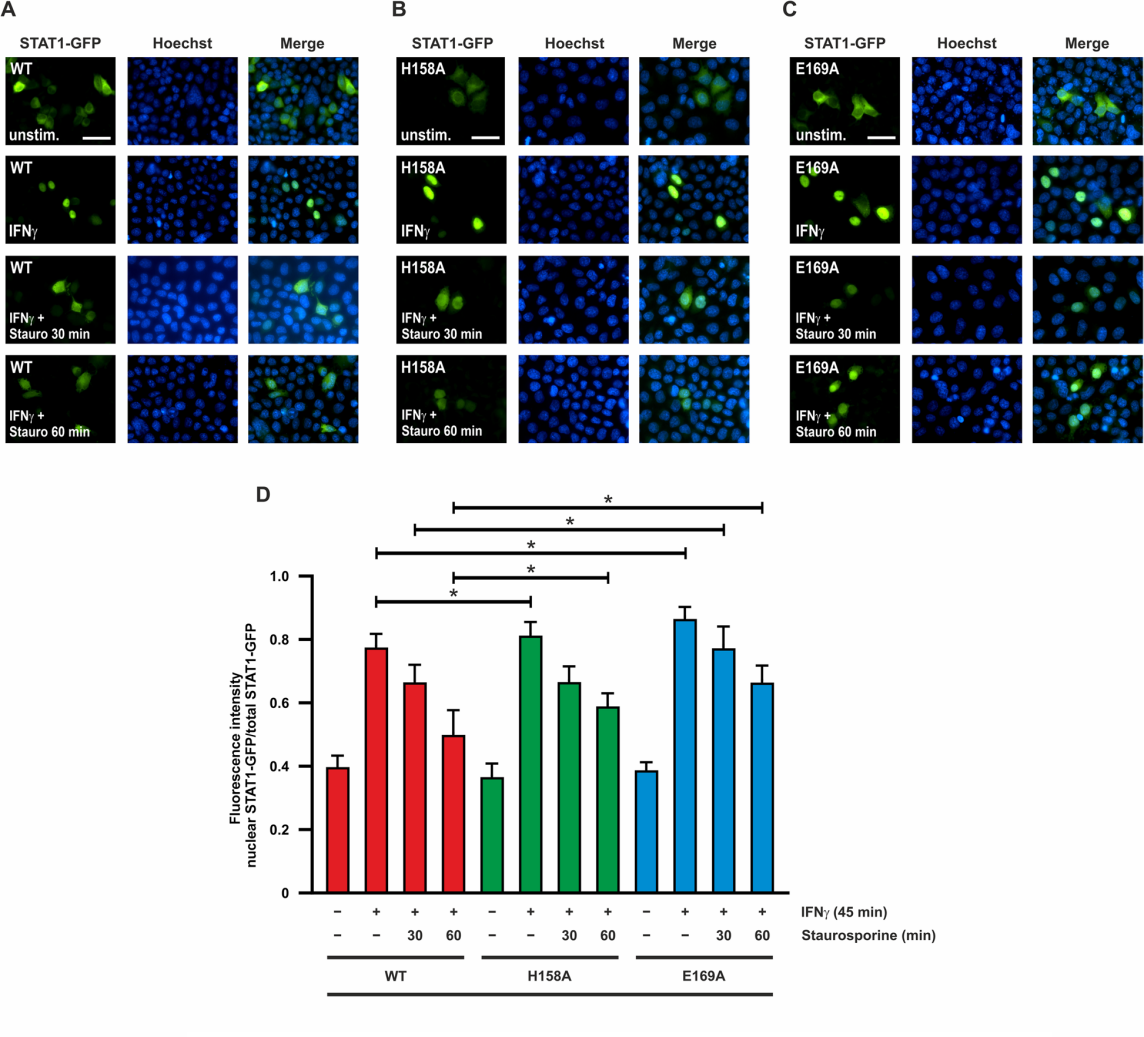
Tyrosine-phosphorylated STAT1-E169A shows prolonged nuclear accumulation. HeLa cells expressing fusion proteins of STAT1-WT (**A**) or the H158A (**B**) and E169A (**C**) mutants were either left unstimulated or stimulated with 50 ng/ml IFNγ for 45 min, this being followed by incubation with staurosporine (1 µM) for the indicated times (scale bar corresponds to 50 µm). **D** Quantification of the results from Fig. 2A–C. STAT1-GFP fluorescence intensity data from n = 20 cells are shown as the ratio of nuclear-to-total STAT1. Asterisks indicate significant differences between the WT protein and the respective mutant

The fluorescence labelling of GFP-tagged STAT1 was additionally used to investigate the kinetics of nuclear accumulation in HeLa cells after stimulation by IFNγ by means of direct fluorescence microscopy. While the cellular distribution in the resting state was unchanged in the two variants compared with the WT variant, STAT1-E169A, once phosphorylated, showed prolonged nuclear accumulation when treated with staurosporine (Fig. 2A-C). This was in contrast to the WT and H158A variants, which regained almost their resting distribution after 60-min exposure to staurosporine. Quantification of nucleocytoplasmic localization showed that these differences were statistically significant (Fig. 2D).

**Fig. 3:**
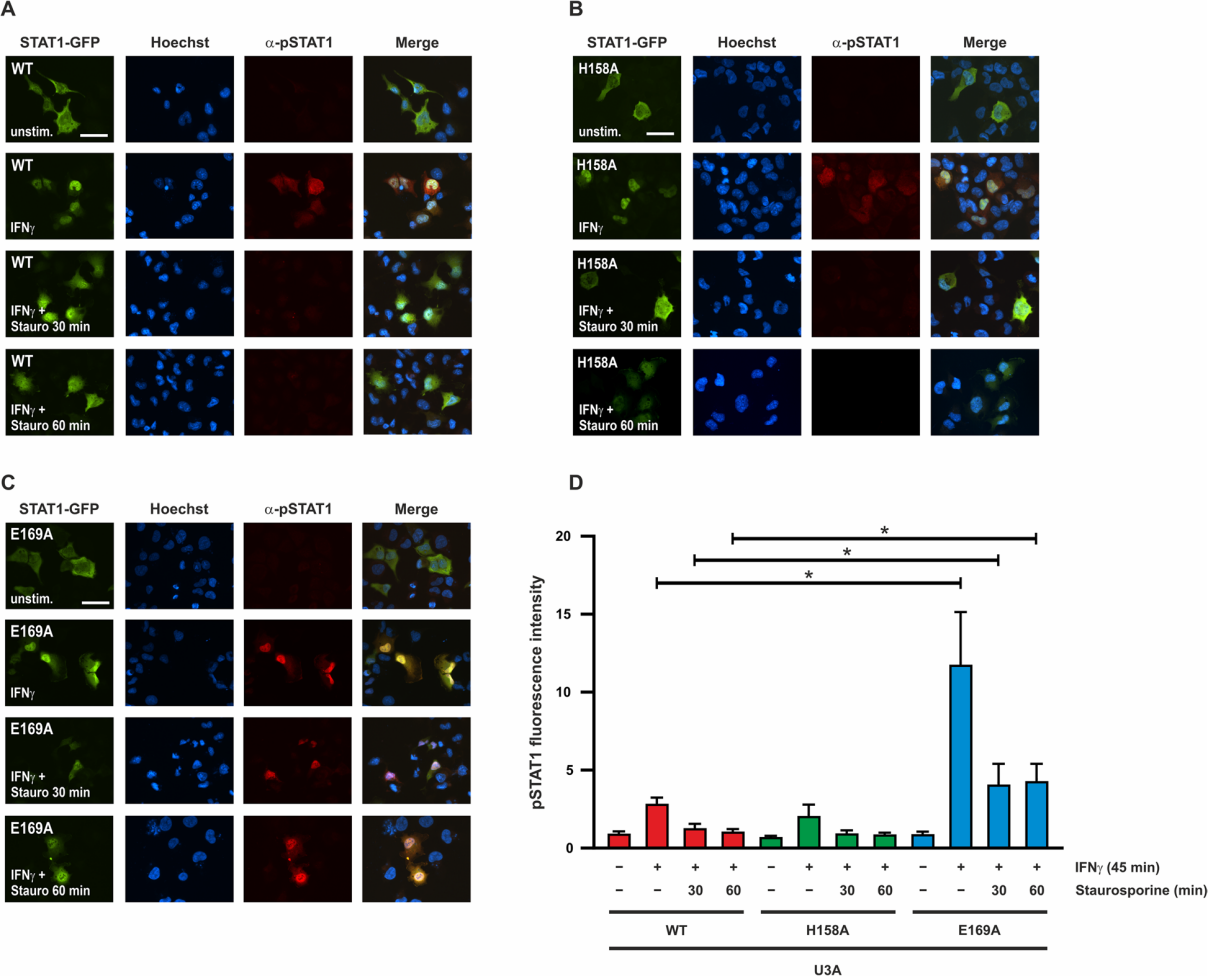
STAT1-E169A retains high levels of tyrosine phosphorylation and nuclear localization. U3A cells expressing fusion proteins of WT (**A**) or mutant (**B**, **C**) STAT1 were either not stimulated or stimulated with 50 ng/ml IFNγ for 45 min before incubation with 1 µM staurosporine for the indicated times (scale bar corresponds to 50 µm). **D** Quantification of the results from Fig. 3A–C. Total fluorescence intensity of tyrosine-phosphorylated STAT1 was measured from n = 20 cells in each experiment. Asterisks indicate significant differences in the tyrosine-phosphorylation level between the WT protein and the E169A mutant

For immunofluorescence experiments, an α-pSTAT1 antibody was used in combination with a secondary antibody coupled to red fluorescent Cy3 dye to examine the cellular distribution of phosphorylated STAT1-GFP in U3A cells after cytokine exposure. Whereas STAT1-WT and H158A largely lost their phosphorylation and nuclear accumulation after staurosporine exposure, the E169 mutant retained a strong immunofluorescence phosphorylation signal that was still detectable after 60 min of kinase inhibition (Fig. 3A-D).

### Accelerated nuclear import of the STAT1-E169A mutant

**Fig. 4:**
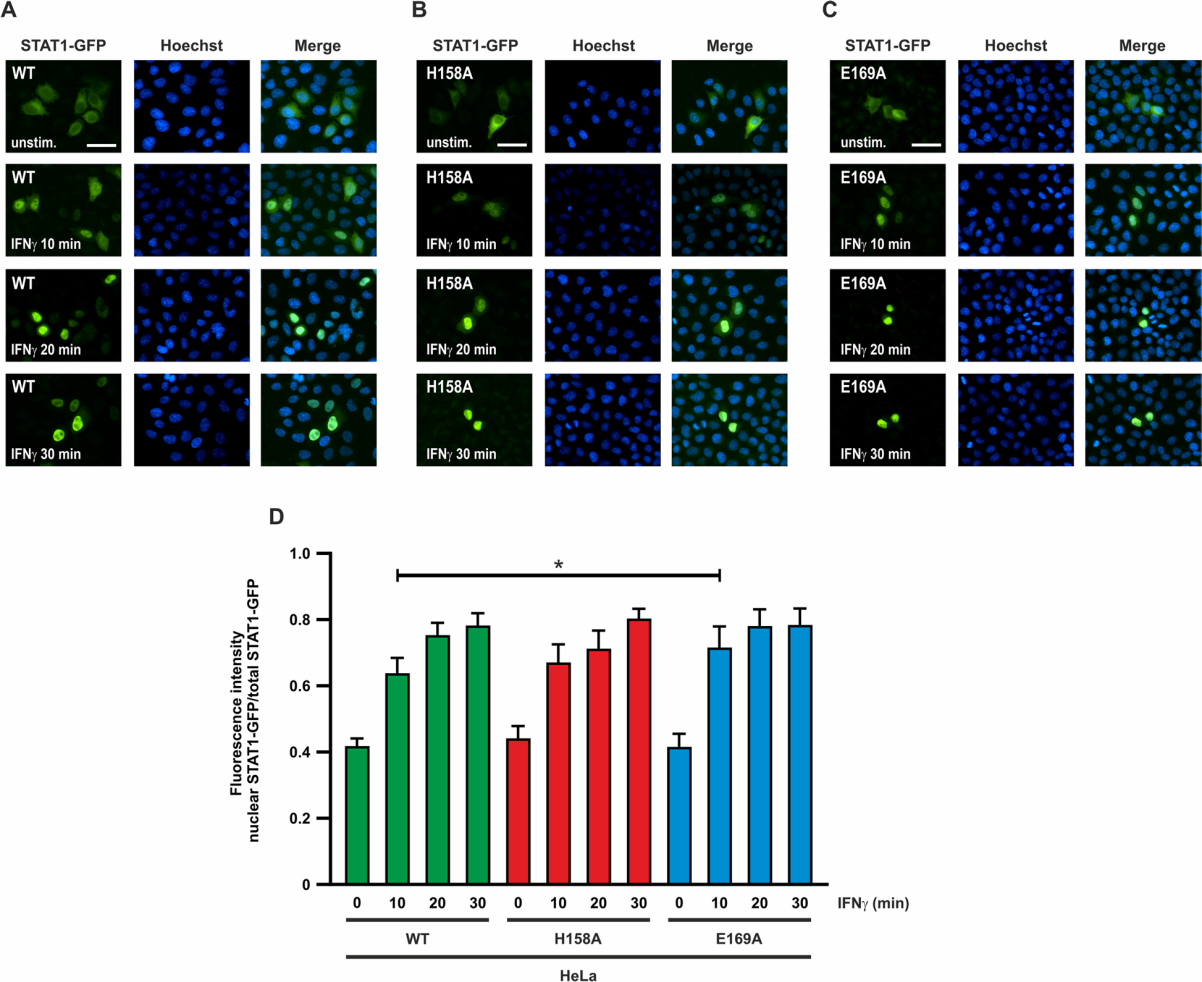
Nuclear accumulation of the STAT1-E169A mutant is accelerated after IFNγ stimulation. HeLa cells expressing fusion proteins of STAT1-WT (**A**) or the H158A (**B**) and E169A (**C**) mutants were either left unstimulated or stimulated with 50 ng/ml IFNγ for the indicated times (scale bar corresponds to 50 µm). **D** Quantification of the results from Fig. 4A–C. STAT1-GFP fluorescence intensity data from n = 20 cells are plotted as the ratio of nuclear-to-total STAT1. Asterisks indicate significant differences between the WT protein and the respective mutant

Given the increased tyrosine phosphorylation and prolonged nuclear accumulation observed for the E169A mutant in the above-mentioned experiments, we again used direct fluorescent microscopy to test the reaction kinetics of nuclear accumulation at early stages of IFNγ stimulation. Prior to cytokine exposure, approximately 40% of the fluorescence intensity was present in the nucleus for all variants, reflecting ground-state import independent of extracellular stimulation. In cells expressing the WT protein or the H158A mutant, nuclear accumulation reached a plateau, where ~ 80% of total STAT1 was located in the nucleus after 30 min of IFNγ treatment (Fig. 4A, B). The E169A mutant also exhibited a significant effect on the localization of the protein in this experiment. In the E169A mutant, the nuclear accumulation reached 70% of the total fluorescence intensity in the nucleus after only 10 min of IFNγ stimulation (Fig. 4C). Thus, nuclear localization of STAT1-E169A is not only prolonged, but also accelerated following IFN stimulation (Fig. 4D).

### STAT1-E169A increases transcriptional activity of target genes

**Fig. 5:**
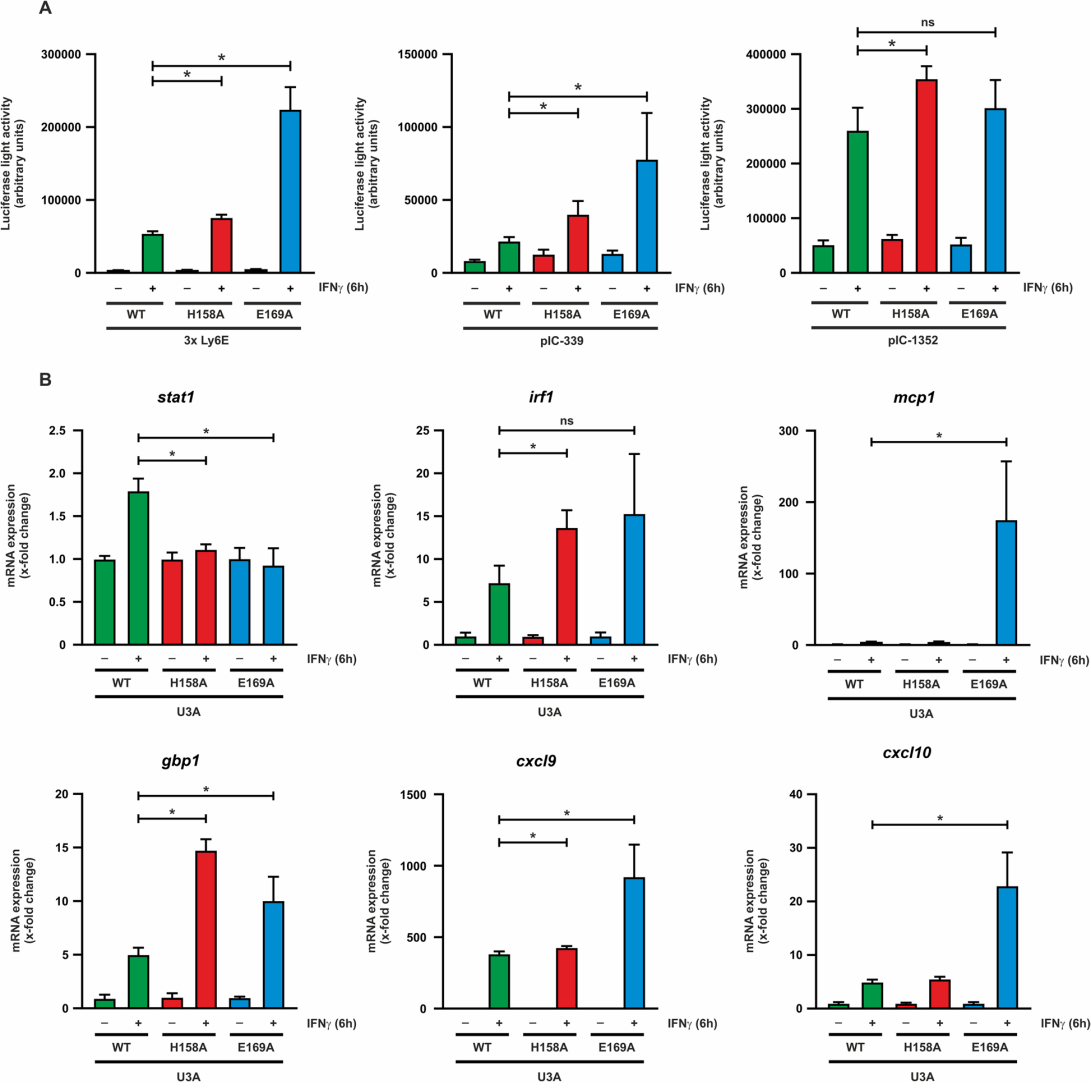
The STAT1-E169A mutation increases transcriptional activity of target genes. **A** U3A cells coexpressing fusion proteins of WT or mutant STAT1 with a luciferase reporter gene coupled to a 3xLy6E, pIC-339 or pIC-1352 promoter were stimulated for 6 h with 50 ng/ml IFNγ. The data are normalized to β-galactosidase coexpression. **B** mRNA levels of endogenous STAT1 target genes in IFNγ-stimulated U3A cells expressing WT or mutant STAT1, obtained by qPCR. Asterisks indicate significant differences between the WT protein and the respective mutant

To determine whether the hyper-phosphorylated E169A interface mutant reflects an overall increase in IFNγ responsiveness, we examined the STAT1 variants for transcriptional activity using a luciferase reporter gene assay. Using both the synthetic promoter 3xLy6E as well as the native promoter sequence pIC-339, we observed a significantly increased gene expression for STAT1-E169A compared with the WT protein (Fig. 5A), and a similar trend for the promotor pIC-1352. These findings were also confirmed by qPCR, measuring mRNA expression of selected endogenous STAT1 target genes. STAT1-E169A showed significantly upregulated expression of all target genes examined, except for *irf1*, which did not reach the significance level. This happened regardless of whether the genes contained a classical, single GAS site (*irf1, gbp1, cxcl9*) or an additional TTC/GAA sequence, termed “one-and-a-half GAS” motif (*mcp1, cxcl10*) (Fig. 5B). Thus, we demonstrated that substitution of the E169 residue leads to STAT1 hyperactivity, which has a direct upregulating effect on the expression of several important ISGs.

### STAT1-E169A binds to DNA with higher affinity

**Fig. 6:**
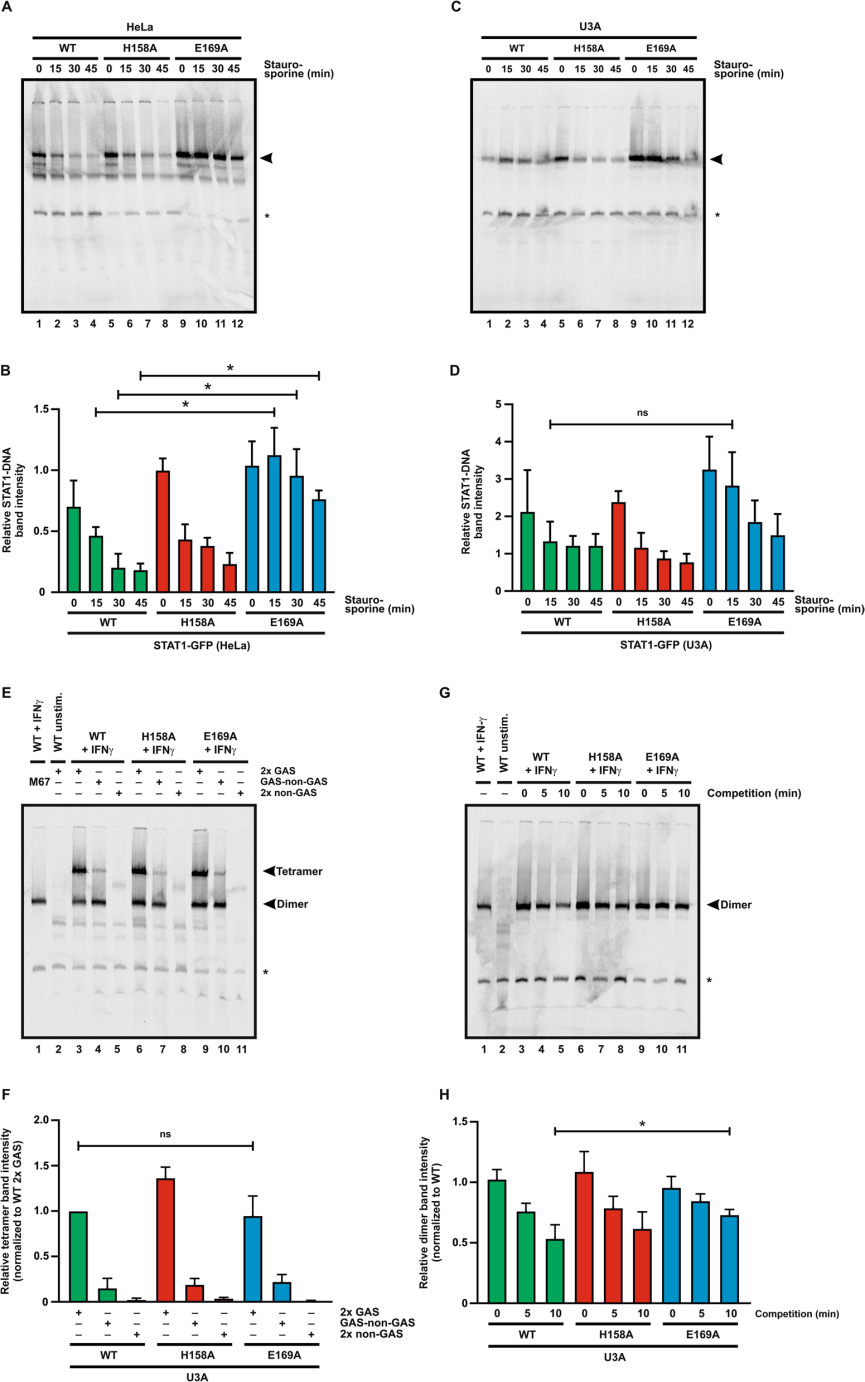
STAT1-E169A binds to DNA with high affinity. HeLa or U3A cells expressing fusion proteins of WT or mutant STAT1 were left unstimulated or stimulated with 50 ng/ml IFNγ for 45 min, and DNA-binding properties were assessed using different DNA probes containing GAS motifs. **A** Representative autoradiogram from HeLa whole cell extracts. HeLa cells were treated with IFNγ for 45 min and subsequently with staurosporine for the indicated times, and whole cell extracts were incubated with M67 probe for 5 min on ice before undergoing electrophoresis. The arrow indicates bands of STAT1-DNA complexes, and the asterisk marks an unspecific band. **B** Quantification of EMSA results from three independent transfection experiments as shown in (**A**). **C, D** Representative autoradiogram from U3A whole cell extracts expressing the indicated untagged STAT1 proteins (**C**), including quantification of results thereof from three independent transfection experiments (**D**). **E** Representative autoradiogram from U3A whole cell extracts. Samples were incubated for 5 min on ice prior to electrophoresis with DNA probes containing either no (2 × non-GAS), one (GAS-nonGAS) or two GAS sites (2 × GAS), as indicated. The asterisk marks a non-specific band. **F** Quantification of EMSA results from three independent transfection experiments as shown in (**E**). **G** Representative autoradiogram from U3A whole cell extracts using a competition EMSA experiment. Samples were incubated with the labeled M67 probe on ice for 30 min and then challenged with a 750-fold excess of unlabeled M67 probe for the indicated times. The asterisk marks a non-specific band. **H** Quantification of EMSA results from three independent transfection experiments as shown in (**G**). In all graphs, asterisks indicate significant differences between the WT protein and the respective mutant

Tyrosine-phosphorylated, DNA-bound STAT1 can be detected by means of an electrophoretic mobility shift assay (EMSA). Whole cell extracts from U3A or HeLa cells expressing the recombinant STAT1 variants were incubated with a [^33^P]-labeled DNA probe containing a single GAS site. The EMSA analysis showed that, upon IFNγ stimulation, greater amounts of the E169A mutant bound the M67 probe and higher levels of STAT1-E169A complexed the single GAS site compared with the WT protein, despite staurosporine treatment (Fig. 6A–D).

Finally, we aimed to further characterize the DNA-binding properties of STAT1-E169A at the molecular level. In the presence of one and a half or two GAS sites in tandem orientation, STAT1-WT utilizes a process called cooperative DNA binding, in which two DNA-bound dimers can interact via their N-termini to improve the stability of the binding complex [31, 32]. By using different DNA probes with a single, double, or no GAS site, we demonstrated that the E169A mutation has no effect on cooperative binding, as no significant differences were observed in DNA-bound complexes compared to the WT protein (Fig. 6E, F).

A third, independent EMSA experiment was performed to assess the dissociation rate of STAT1-E169A from the DNA in a competition assay. In this assay, whole cell extracts from transfected U3A cells were first incubated with labeled M67 probe, and the resulting protein-DNA complexes were subsequently challenged with a 750-fold molar excess of unlabeled M67 competing with the STAT1 binding. In the presence of unlabeled M67, STAT1-WT rapidly dissociated from the radiolabeled probe and was no longer detectable. In contrast, the E169A mutant displayed a significantly increased binding affinity and largely resisted competition (Fig. 6G, H).

## Discussion

Like other CCD GOF mutants described in the literature, the present study shows that substitution of alanine for glutamic acid in residue 169 results in a hyper-phosphorylated phenotype with increased transcriptional activity. The same mutation has been associated with an increased susceptibility to fungal infections in two related patients [26]. Although the previously investigated STAT1 mutants with a similar hyperactive phenotype such as T385A or F274W disrupt the formation of an antiparallel dimer, none of them could be associated with an increase in DNA-binding affinity [22, 33]. Therefore, the altered DNA-binding properties we observed for E169A suggest a different and unique molecular mechanism behind the hyperactivity of the mutant.

The conventional, reciprocal CCD-DBD interaction stabilizes antiparallel dimers and, when mutated, is responsible for most clinically relevant phenotypes [18–23]. Although the amino acid residue E169 is also located in the CCD, it does not contribute to this interaction. Instead of contacting the DBD of another protomer, it comes into proximity with an N-terminal domain, as shown in the crystal structure of the antiparallel tetramer [25].

**Fig. 7:**
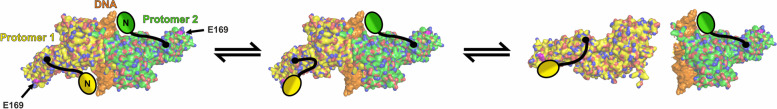
A novel mechanism for the dissociation of STAT1 from DNA through auto-inhibition. Surface representation of a tyrosine-phosphorylated parallel STAT1 dimer (yellow + green) binding to DNA (orange). The position of the respective N-terminal domains (N) is approximated. The critical amino acid position E169 (magenta) in the CCD is marked with arrows. According to the model, binding of the N-terminus to the E169 residue of its own core fragment facilitates dissociation from DNA

Our findings suggest that the amino acid residue E169 is probably involved in a process, which controls the inactivation of the STAT1 signaling pathway. In the WT molecule, interaction of the N-terminus of a DNA-bound dimer with the CCD of its own or another protomer ultimately helps to release STAT1 from the DNA to deactivate the signaling cascade. This hypothesis may explain why the E169A mutant showed a reduced rate of dissociation from the DNA in a competition assay, whereas other CCD mutations had no direct effect on DNA-binding affinity, although they share the same phenotype in many other aspects, as explained above. Therefore, we propose a previously unknown mechanism of STAT1 auto-inactivation mediated by intramolecular binding of the N-terminus of a DNA-bound STAT1 dimer to the E169 residue in the CCD of the core fragment (Fig. 7). Mutation of the E169 residue disrupts this interaction and results in a hyperactive phenotype, which upregulates transcription of a variety of target genes involved in antimicrobial immune reactions. Alternatively, another inhibitory mechanism could be feasible, in which the N-terminus binds to the CCD of a free protomer that is not involved in DNA binding.

The proposed involvement of the N-termini in the inactivation of the STAT1 signaling pathway is not an entirely new concept. Previous studies from the laboratory of Darnell have described a model, wherein the interaction between the two N-termini stabilizes the dimer while it undergoes a conformational shift from a parallel to an antiparallel orientation [9, 10]. This change in the dimer conformation allows for the subsequent dephosphorylation and inactivation of the transcription factor. Based on our data, we suggest an alternative model for the inactivation of DNA-bound dimers, which relies on the interaction of the N-terminal domain with the coiled-coil domain to terminate the signal.

Fluorescence recovery after photobleaching (FRAP) of a GFP-fusion protein with the N-terminal deletion mutant (STAT1-ΔN-GFP) showed increased intracellular mobility exclusively in IFNγ-stimulated cells, but not in unstimulated cells, compared to the WT, as demonstrated by the Vinkemeier laboratory [34]. Their observation of a higher nuclear mobility of tyrosine-phosphorylated STAT1-ΔN compared to the full-length protein is in line with our hypothesis that the N-terminus contributes to DNA binding. According to our model, the intramolecular binding of the N-terminus to the E169 residue in the CCD prevents the N-terminus from participating in the DNA binding. This weakens the stability of the DNA-bound dimer, and thus, facilitates the dissociation of the dimer from DNA. Furthermore, STAT1 is also prevented to bind to DNA in its monomeric form.

In addition, the E169 residue may play a vital role in the stabilization of the inactive, antiparallel STAT1 tetramer, where it binds to an N-terminal domain. In the crystal structure of the tetramer, we noticed two slightly different orientations of the respective N-termini in relation to the CCD, which leads to dissimilar interaction partners for the E169 residue, depending on the observed protomer (Fig. 1C, D). In a previous publication, we identified N-terminal point mutations, which displayed altered DNA binding, leading to a hyperactive phenotype [35].

The tetrameric complex probably acts as a cellular reservoir for non-DNA-bound STAT1 to prevent abnormal pathway activation. We observed that disruption of this interaction interface leads to hyper-phosphorylation of STAT1 and accelerated nuclear build-up, along with prolonged nuclear retention and upregulated target gene expression. Based on these results, we hypothesize that the E169A mutant impairs the formation of the tetrameric structure, thereby shifting the equilibrium towards the transcriptionally active parallel dimer conformation.

In summary, our mutational analysis revealed an unknown interface between the CCD and the N-terminus of STAT1, mediated by the critical glutamic acid residue at position 169. Disruption of this interaction inhibits pathway termination and increases transcription of target genes in an aberrant manner. This finding suggests a novel auto-inhibitory mechanism for STAT1, which involves the previously unidentified N-terminal:CCD interaction to assist in the release of a STAT1 dimer from the DNA. Given that the E169A substitution mutation has been described in two patients diagnosed with chronic mucocutaneous candidiasis, it is evident that the proposed auto-inhibitory mechanism of STAT1 inactivation has great clinical significance.

## Supplementary Information

**Additional file 1: Supplemental Fig. 1.** Uncropped, original Western blot data using α-pSTAT1 and α-STAT1 antibodies, corresponding to Fig. 1.

## Data Availability

The datasets generated during and/or analyzed during the current study are available from the corresponding author on reasonable request.
