# Supplementary figures and images for: A novel interface between the N-terminal and coiled-coil domain of STAT1 functions in an auto-inhibitory manner
Source: Cell Commun Signal. 2023 Jul 10;21:170. doi: 10.1186/s12964-023-01124-1 (PMC10331961; doi:10.1186/s12964-023-01124-1)

STAT1-GFP Blot 1

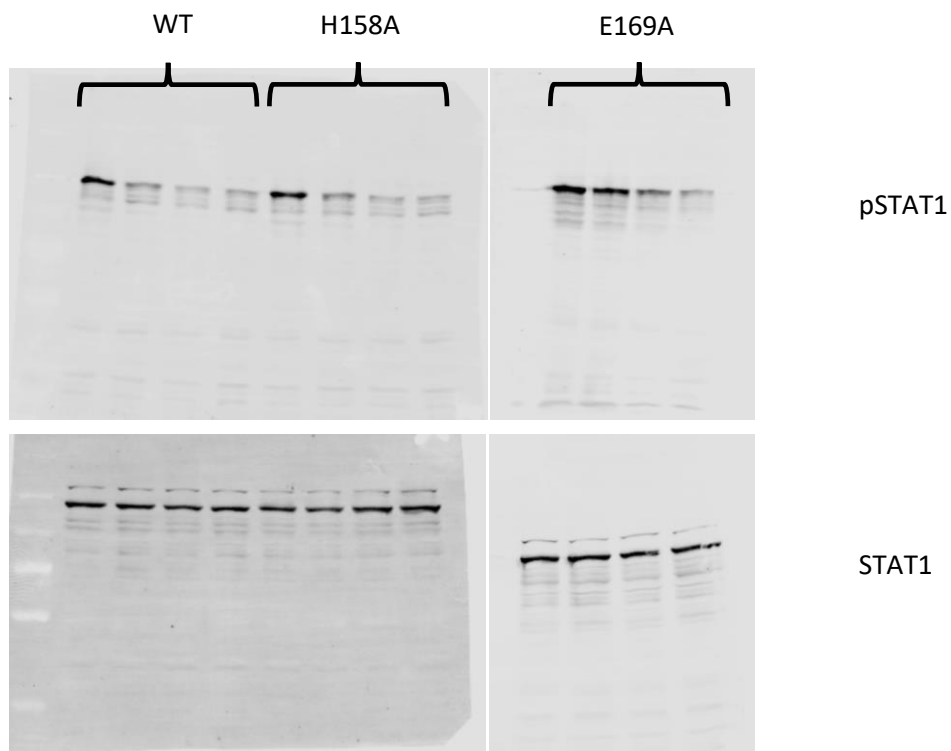

STAT1-GFP Blot 2

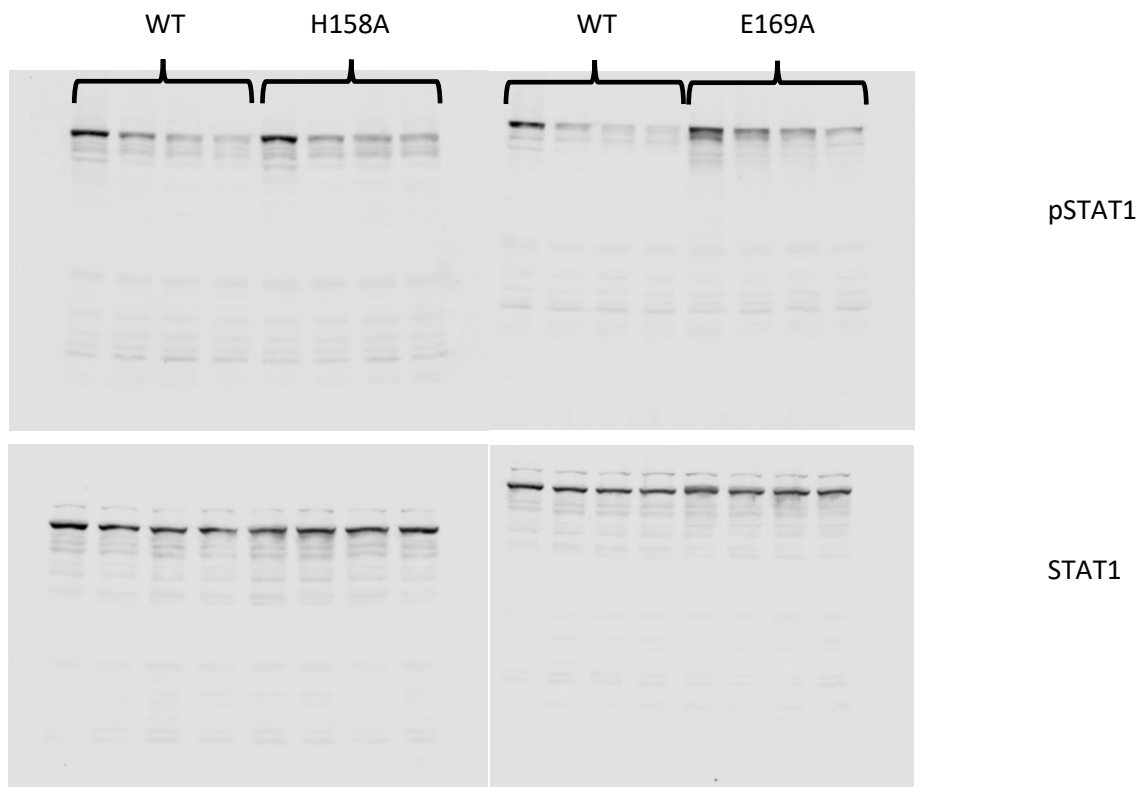

STAT1-GFP Blot 3

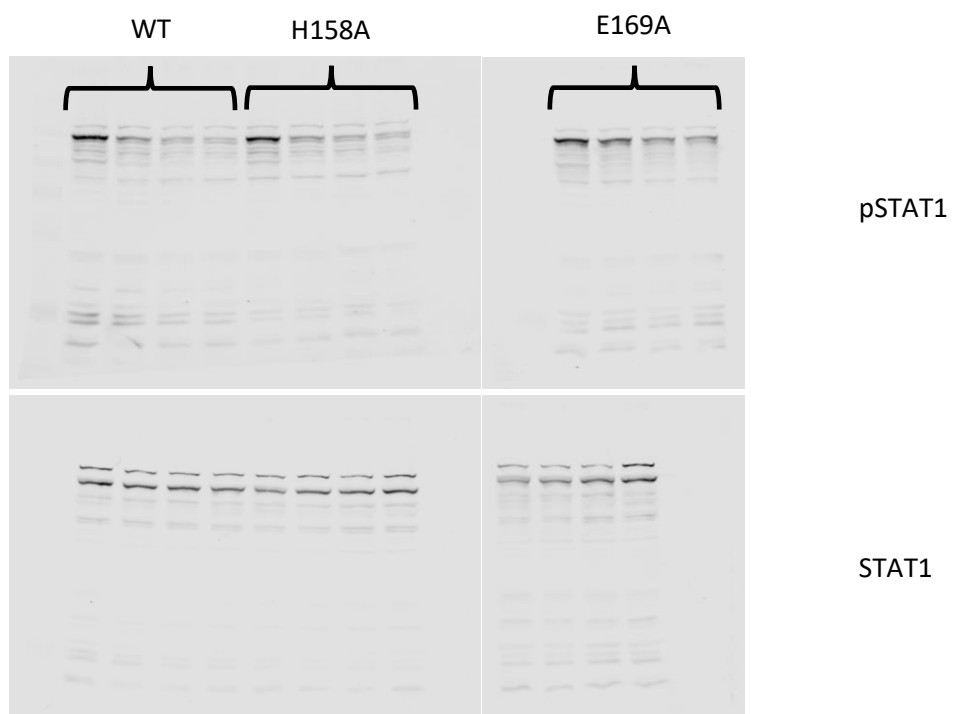

STAT1 pcDNA Blot 1

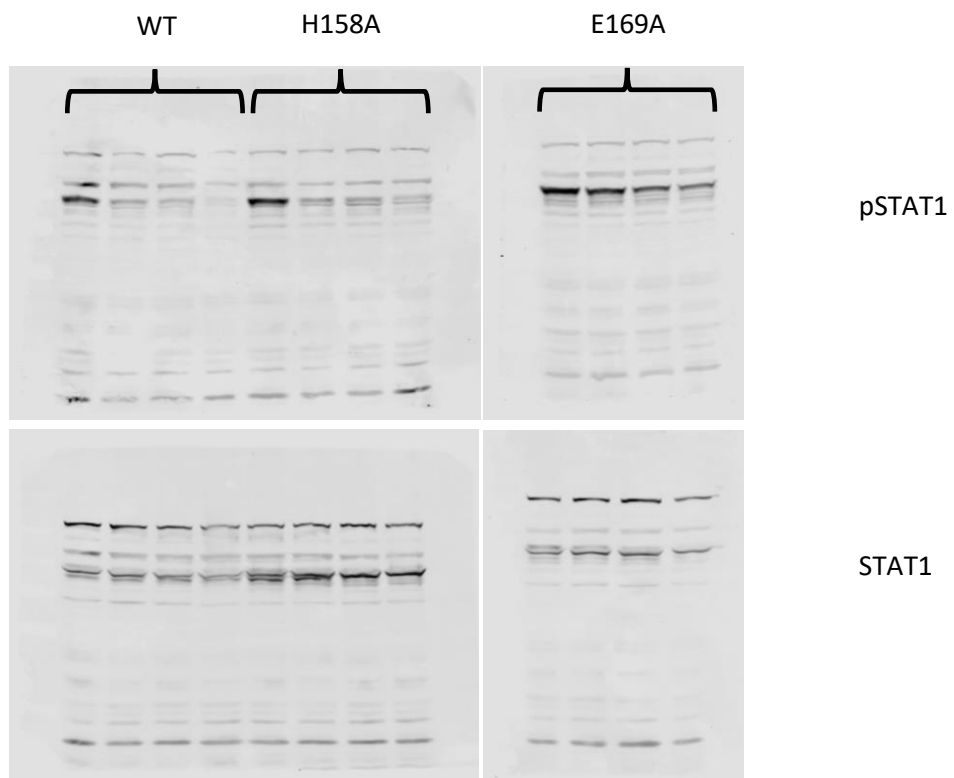

STAT1 pcDNA Blot 2

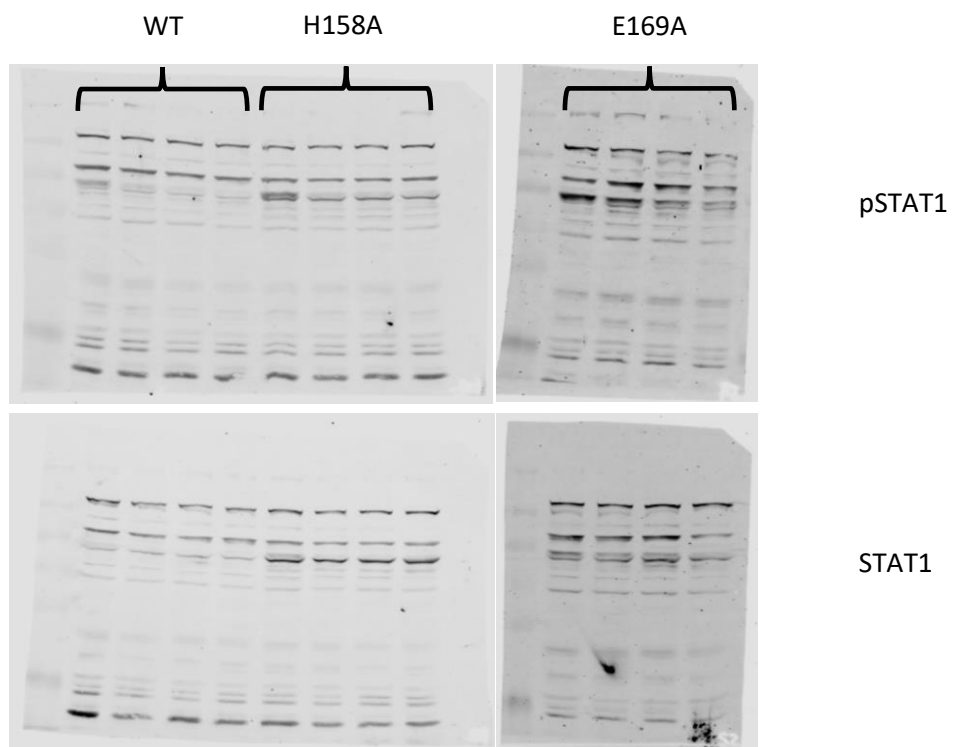

STAT1 pcDNA Blot 3

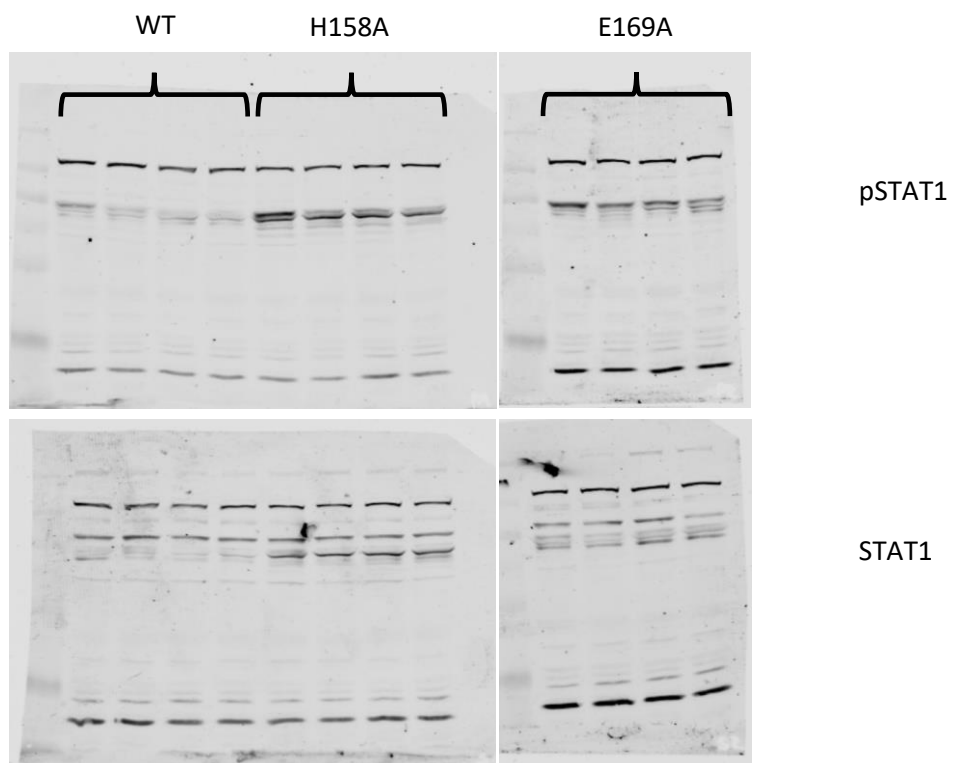

Supplement: Supplementary file 2 — Additional file 1: Supplemental Fig. 1. Uncropped, original Western blot data using α-pSTAT1 and α-STAT1 antibodies, corresponding to Fig. 1. [file 12964_2023_1124_MOESM1_ESM.pdf]
